# Pregnancy and delivery in woman with implantable cardioverter-defibrillator: what we should know

**DOI:** 10.11604/pamj.2018.30.236.9995

**Published:** 2018-07-30

**Authors:** Mohamed Amine Bouslama, Fehmi Ferhi, Faten Hacheni, Kaabia Ons, Khlifi Abdeljelil, Khaled Ben Jazia, Hedi Khairi

**Affiliations:** 1 Anesthesia Department, CHU Farhat Hached, Sousse, Tunisia; 2 Obstsetric Department, Farhat Hached Hospital, Sousse, Tunisia

**Keywords:** Pregnancy, implantable cardioverter-defibrillator, anesthesia

## Abstract

We report the observation of a 25-year-old pregnant patient of 39 weeks of amenorrhea proposed for elective cesarean section. This patient suffers from hypertrophic cardiomyopathy since the age of 12. She has an implantable cardioverter defibrillator (ICD). The peculiarities of the ICD in the parturient and the perioperative management of the patient are being reported in this paper.

## Introduction

An implantable cardioverter-defibrillator (ICD) improves survival in patients with life-threating arryhtmias. The indications for ICD implantation include younger age patients with congenital heart disease reaching a reproductive age. Severe ventricual arrhythmias can be triggered during pregnancy as a result of physiologic modifications [**1**]. There are a few studies of pregnancy with ICD managed and there are actually no guidelines for pregnancy and delivery in patients with an ICD.

## Patient and observation

**Figure 1:**
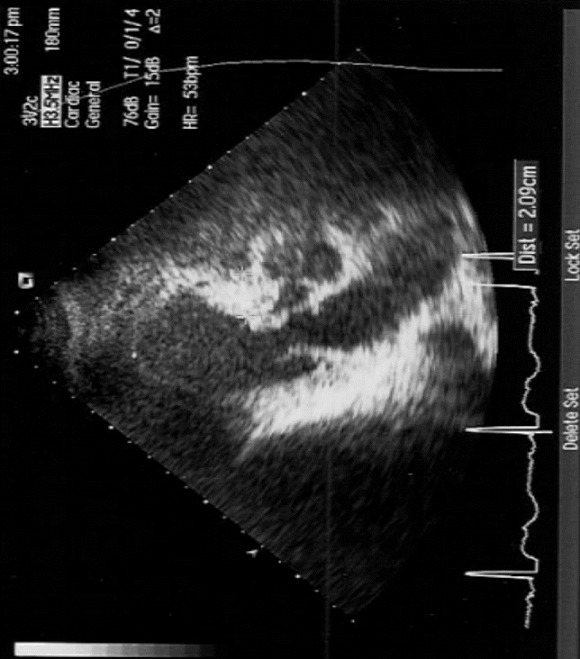
Transthoracic ultrasonography showed an asymmetrically overdeveloped left ventricle

We report the case of a 25-year-old woman (164cm, 84kg, G2 P1). She was referred to the tertiary care level delivery unit of Farhat Hached University Hospital for a scheduled c-section at 37 weeks of gestation. The medical history of the parturient notes a hypertrophic cardiomyopathy diagnosed at the age of 12 in a context of a repeated shortness of breath. She has been put under bisprolol 2.5 mg/day. The evolution of the illness was marked by the occurrence of two episodes of syncope at the age of twenty. The rhythmologic exploration has highlighted severe and paroxysmal ventricular rhythm disorders indicating the placement of a double room implantable cardioverter defibrillator (ICD). Afterwards, the evolution was uneventful. The current pregnancy was spontaneous and well followed by the obstetrician and the cardiologist (monthly consultation). No cardiovascular or obstetric complication was noted. Bisprolol dose was doubled throughout the pregnancy. The patient presented for an elective cesarean section at 39 SA. Clinical examination showed 86 heartbeats per minute, blood pressure of 110/80 mm Hg and a respiratory rate of 18 cycles/min. A transthoracic ultrasonography showed an asymmetrically overdeveloped left ventricle (LV), an inter ventricular septum at 28 mm, a Left ventricular ejection fraction of 80%, an intra LV gradient of 10 mm Hg, a disorder of the compliance of the LV with high filling pressures of the LV, a non-dilated left atrium, and not distended right cavities with normal systolic function of the right ventricle (Figure 1). Anesthetic examination shows a good venous capital and no evidence for oral intubation difficulty.

The patient was sent on the eve of the caesarean section to her cardiologist who decided the deactivation of the defibrillator given the risk of inadvertent activation during the scheduled surgery. In the operating room, the patient received invasive monitoring of her blood pressure, her electrocardiogram, her pulse oxygen saturation measurement and her expired CO2 pressure. The venous infusion during the surgery was established with a 16 G peripheral branule in the back of the left hand prefilled with 500ml of saline. The patient had a rachi-analgesia at the level of L4 - L5 with 200μg of morphine. The induction was with a rapid sequence with 3 mg/kg propofol, 1 mg/kg of suxamethonium and 0.5μg/kg of remifentanil. Oral intubation was performed without incident. The anesthesia was performed by of sevoflurane and of remifentanil at a dose of 0.05g/kg/min. An antibiotic prophylaxis with 2g of Cefazolin was administered after the clamping of the umbilical cord. Ten minutes after the anesthetic induction occurred the fetal extraction of a female newborn of 2900 g weight and an Apgar score of 8/9/10. Five IU of oxytocin were slowly intravenously injected then, followed after the delivery by a dose of 20 IU of oxytocin for 4 hours. During the surgery the hemodynamic parameters were stable (no heart arhythmia) authorizing the awakening and extubation of the patient on the operating table. The patient was transferred in the resuscitation care unit. She received a multimodal analgesia. The ICD has been reactivated 6 hours after the end of the intervention. Postoperative outcomes were simple: in particular, no cardiac rhythm disorder, or heart failure. The patient was released 3 days after her delivery. She was referred to her cardiologist for a further adjustment of her drugs.

## Discussion

The cardiac pathology represents 1% of all the complications in pregnant women. Among them, hypertrophic cardiomyopathy (HCM) remains a serious pathology with potentially deadly complications. The HCM is a dominant autosomic congenital heart disease with a low penetrance and an expression characterized by a remodeling of the myocardial tissue of the right ventricule with an anarchic provision of hypertrophied myocardial fibers, and the emergence of a significant fibrosis that exposes the patients to the risk of sudden death [1]. The physiological changes during pregnancy, such as the increase in the maternal blood volume, the elevation of the heart rate, and the elevation of the cardiac output, are simulated physiological stress tests that may be the cause of cardiac rhythm disorders in patients with heart arhythmogenic diseases such as hypertrophic cardiomyopathy (HCM) [2]. The implantable cardioverter defibrillator (ICD) has emerged as a preventive treatment in patients at high risk of sudden death by a serious heart rhythm disorder [3]. This new therapy has transformed the prognosis of arhythmogenic heart diseases, often diagnosed at a young age resulting in an increase in the number of patients reaching the age of procreation [4].

It has been shown that the ICD is not a contraindication to pregnancy but it cannot fully prevent from the onset of a threatening cardiac rhythm disorder during pregnancy. In fact, the ICD-carrier parturients are not fully protected against all maternal and fetal complications that may jeopardize their prognosis [5]. That's why a careful monitoring of the HCM patients is a necessary to detect and prevent the slightest cardiac complication. No study has shown an elevation of the risk of cardiac events secondary to a dysfunction of the ICD during pregnancy [6, 7]. However a telemetric consultation prior to the conception and thus the pregnancy is necessary to check the proper functioning of the enclosure settings: detection, threshold and stimulation impedance [5]. There are no clear recommendations regarding the deactivation of the ICD during pregnancy. After deactivating the ICD, the occurrence of a ventricular rhythm disorder may be deleterious to the fetus through a low placental perfusion due to a maternal arterial hypotension. On the other hand, even a low energy shock, if transferred in utero, can still be harmful for the fetus, in case of the activation of the ICD during the pregnancy. Thus, it is recommended to deactivate the ICD during the delivery which is subject to specific maternal and fetal cardiac monitoring [7].

Other complications can occur during pregnancy and delivery such as the migration of the remote control or the necrosis of maternal tissues [8]. The management of pregnant patients with ICD should be multidisciplinary associating an obstetrician, a cardiologist and an anesthesiologist. The use of beta blockers significantly reduced the risk of cardiac arrhythmia in case of arhythmogenic heart disease. These drugs are strongly recommended in HCM parturient patient during pregnancy despite their hypothetic risk of bradycardia and hypoglycemia in the newborn. In case of an emergency caesarian section delivery in a parturient with an active ICD, it is recommended to use during surgery a bipolar scalpel after the placement of a magnet [9].

Vaginal birth remains the gold standard concerning the mode of delivery for parturient women living with a heart disease and all ICD carriers [10]. Epidural analgesia is recommended to reduce the secondary sympathetic reaction to the painful stimulation of the uterine contractions [3]. However the anesthetic management is dependent on the evolution of the underlying heart disease. Our patient has benefited from a general anesthesia due to the scalability of her hypertrophic cardiomyopathy and the major risk of hemodynamic alteration during the surgery. The hemodynamic objective during the surgical procedure regardless to the anesthetic technique is to prevent any low maternal blood volume and any hypotension that may result in arrhythmia in the parturient and a low placental perfusion leading to acute fetal suffering. After the delivery, a close monitoring in intensive care unit is recommended. In fact, the risk of cardiac rhythm disorders persists during this period. The ICD must be reactivated and a telemetric consultation is required.

## Conclusion

It is obvious that the implantable cardioverter defibrillator is not a contraindication to pregnancy. ICD carrier parturients require a careful medical follow-up and a multidisciplinary management, in order to avoid some complications that could alter the maternal and fetal prognosis.

## Competing interests

The authors declare no competing interest.
